# Supplementary material for: From sequence to enzyme mechanism using multi-label machine learning
Source: BMC Bioinformatics. 2014 May 19;15:150. doi: 10.1186/1471-2105-15-150 (PMC4229970; doi:10.1186/1471-2105-15-150)
Supplement: Additional file 2 — Java code of ml2db. Additional file ml2db_code.tar.gz contains the Java source code to run the multi-label machine learning experiments and save the results to database. The code’s Javadoc is included. [file 1471-2105-15-150-S2.zip › additional file 2/ml2db/ml2db/doc/mulan/classifier/MultiLabelLearner.html]

MultiLabelLearner


JavaScript is disabled on your browser.


- Overview
- Package
- Class
- Use
- Tree
- Deprecated
- Index
- Help

- Prev Class
- Next Class

- Frames
- No Frames

- All Classes

- Summary:
- Nested |
- Field |
- Constr |
- Method

- Detail:
- Field |
- Constr |
- Method


mulan.classifier

## Interface MultiLabelLearner

- All Known Implementing Classes:
  :   AdaBoostMH, BinaryRelevance, BPMLL, BRkNN, CalibratedLabelRanking, ClassifierChain, ClusteringBased, EnsembleOfClassifierChains, EnsembleOfPrunedSets, EnsembleOfSubsetLearners, ExampleBasedFMeasureOptimizer, HMC, HMCNode, HOMER, IBLR\_ML, IncludeLabelsClassifier, LabelPowerset, LabelsetPruning, Meta, MetaLabeler, MLkNN, MLPTO, MMPLearner, MultiClassLearner, MultiLabelKNN, MultiLabelLearnerBase, MultiLabelMetaLearner, MultiLabelStacking, OneThreshold, PPT, PrunedSets, RAkEL, RAkELd, RCut, SCut, SubsetLearner, ThresholdPrediction, TransformationBasedMultiLabelLearner

  ---

    

  ```
  public interface MultiLabelLearner
  ```

  Common root interface for all multi-label learner types.

  Author:
  :   Jozef Vilcek

- - ### Method Summary

    Methods

    | Modifier and Type | Method and Description |
    | --- | --- |
    | `void` | `build(MultiLabelInstances instances)` Builds the learner model from specified `MultiLabelInstances` data. |
    | `boolean` | `isUpdatable()` Returns value indicating if learner is updatable, so if learner is able to perform on-line learning. |
    | `MultiLabelLearner` | `makeCopy()` Creates a deep copy of the given learner using serialization. |
    | `MultiLabelOutput` | `makePrediction(weka.core.Instance instance)` Returns the prediction of the learner for a given input `Instance`. |
    | `void` | `setDebug(boolean debug)` Sets whether debugging information should be output by the model |

- - ### Method Detail


    - #### isUpdatable

      ```
      boolean isUpdatable()
      ```

      Returns value indicating if learner is updatable, so if learner is able to
      perform on-line learning. The fact if learner is updatable or not influences
      the behavior of `build(MultiLabelInstances)` method.  
      When `false` is returned, each call of the
      `build(MultiLabelInstances)` will initialize the learner from
      the scratch, removing any potential knowledge built by previously entered training data.
      When `true` is returned, then on the first call of the
      `build(MultiLabelInstances)` the learner is initialized
      with the passed training data. All other calls contribute to the existing learner's
      model with new data.

      Returns:
      :   `true` if learner is updatable (on-line), `false` otherwise.


    - #### build

      ```
      void build(MultiLabelInstances instances)
                 throws java.lang.Exception,
                        InvalidDataException
      ```

      Builds the learner model from specified `MultiLabelInstances` data.
      Sequential calls to this method either re-build the learners model with new data
      (off-line learner) or contribute to the existing model with new data (on-line learner).
      The behavior is determined by the outcome of `isUpdatable()` method.

      Parameters:
      :   `instances` - set of training data, upon which the learner model should be built

      Throws:
      :   `java.lang.Exception` - if learner model was not created successfully
      :   `InvalidDataException` - if specified instances data is invalid or not supported by the learner

      See Also:
      :   `isUpdatable()`


    - #### makeCopy

      ```
      MultiLabelLearner makeCopy()
                                 throws java.lang.Exception
      ```

      Creates a deep copy of the given learner using serialization.

      Returns:
      :   a deep copy of the learner

      Throws:
      :   `java.lang.Exception` - if an error occurs while making copy of the learner.


    - #### makePrediction

      ```
      MultiLabelOutput makePrediction(weka.core.Instance instance)
                                      throws java.lang.Exception,
                                             InvalidDataException,
                                             ModelInitializationException
      ```

      Returns the prediction of the learner for a given input `Instance`.

      Parameters:
      :   `instance` - the input given to the learner in the form of `Instance`

      Returns:
      :   a prediction of the learner in form of `MultiLabelOutput`.

      Throws:
      :   `java.lang.Exception` - if an error occurs while making the prediction.
      :   `InvalidDataException` - if specified instance data is invalid and can not be processed by the learner
      :   `ModelInitializationException` - if method is called before `build(MultiLabelInstances)`


    - #### setDebug

      ```
      void setDebug(boolean debug)
      ```

      Sets whether debugging information should be output by the model

      Parameters:
      :   `debug` -


- Overview
- Package
- Class
- Use
- Tree
- Deprecated
- Index
- Help

- Prev Class
- Next Class

- Frames
- No Frames

- All Classes

- Summary:
- Nested |
- Field |
- Constr |
- Method

- Detail:
- Field |
- Constr |
- Method
